# Intravenous high-dose anakinra drops venous thrombosis and acute coronary syndrome in severe and critical COVID-19 patients: a propensity score matched study

**DOI:** 10.1038/s41598-024-62079-y

**Published:** 2024-05-29

**Authors:** Ramazan Çakmak, Servet Yüce, Mustafa Ay, Muhammed Hamdi Uyar, Muhammed İkbal Kılıç, Murat Bektaş

**Affiliations:** 1 Division of Endocrinology and Metabolism, Department of Internal Medicine, Istinye University, Istanbul, Turkey; 2 Department of Public Health and Biostatistics, Istanbul Faculty of Medicine, Istanbul, Turkey; 3 Aksaray University, Aksaray Training and Research Hospital, Aksaray, Turkey; 4 Department of Internal Medicine, Aksaray Training and Research Hospital, Aksaray, Turkey; 5 Division of Rheumatology, Department of Internal Medicine, Aksaray Training and Research Hospital, Yeni Sanayi Street, Merkez, 68200 Aksaray, Turkey; 6 Division of Rheumatology, Department of Internal Medicine, Istanbul Aydın University, Istanbul, Turkey

**Keywords:** Anakinra, COVID-19, Thrombosis, Inflammasome, Hyperinflammation, Acute coronary syndrome, Immunology, Microbiology, Rheumatology

## Abstract

In our study, we aimed to evaluate the effect of high-dose intravenous anakinra treatment on the development of thrombotic events in severe and critical COVID-19 patients. This retrospective observational study was conducted at a tertiary referral center in Aksaray, Turkey. The study population consisted of two groups as follows; the patients receiving high-dose intravenous anakinra (anakinra group) added to background therapy and the patients treated with standard of care (SoC) as a historical control group. Age, gender, mcHIS scores, and comorbidities such as diabetes mellitus, hypertension, and coronary heart disease of the patients were determined as the variables to be matched. We included 114 patients in SoC and 139 patients in the Anakinra group in the study. Development of any thromboembolic event (5% vs 12.3%, p = 0.038; OR 4.3) and PTE (2.9% vs 9.6%, p = 0.023; OR 5.1) were lower in the Anakinra group than SoC. No patient experienced cerebrovascular accident and/or clinically evident deep venous thrombosis both in two arms. After 1:1 PS matching, 88 patients in SoC and 88 patients in the Anakinra group were matched and included in the analysis. In survival analysis, the development of any thromboembolic event, pulmonary thromboembolism, and acute coronary syndrome (ACS) were higher in SoC compared to Anakinra. Survival rate was also lower in patients with SoC arm than Anakinra in patients who had any thromboembolic event as well as ACS. In our study, the development of thrombosis was associated with hyperinflammation in patients with severe and critical COVID-19. Intravenous high-dose anakinra treatment decreases both venous and arterial events in patients with severe and critical COVID-19.

## Introduction

Coronavirus-19 (COVID-19) is an emerging infectious disease caused by severe acute respiratory syndrome coronavirus 2 (SARS-CoV-2) and affects many organs mainly upper and lower respiratory tracts. Disease severity of COVID-19 ranges from asymptomatic and/or mild symptoms to potential life-threatening disease including acute respiratory distress syndrome (ARDS), multi-organ failure, and even death. Several risk factors such as male gender, advanced age, some comorbidities including diabetes mellitus (DM), hypertension (HT) and coronary heart disease (CHD), and immunosuppressive treatment were described for the development of poor prognosis as well as severe course in COVID-19^1^.

Hyperinflammation (cytokine storm) is one of the main features of severe disease in COVID-19 and is also closely associated with poor outcomes including ARDS, the need for oxygen therapy, and higher mortality^2^. Several immunomodulatory treatments such as corticosteroids, baricitinib, anakinra, and tocilizumab were found to be effective in COVID-19 patients with signs of hyperinflammation^3–6^.

In addition to cytokine storm, some patients suffer from thrombotic events including acute coronary syndrome (ACS), cerebrovascular accident (CVA), and venous thromboembolism (VTE) such as deep vein thrombosis (DVT) and pulmonary thromboembolism (PTE) during the course of COVID-19^7^. Thereby, prophylactic use of anticoagulant and/or antiaggregant therapies were applied especially in hospitalized COVID-19 patients in daily practice^8^. However, some studies have shown reduced mortality and also the development of thromboembolic events with prophylactic use of anticoagulant therapy^9–11^, there are conflicting results with the benefit of anticoagulant therapy in terms of development of mortality and/or thrombosis^12^. Moreover, it is not known whether immunomodulatory therapy reduces thromboembolic events in patients with severe COVID-19.

In our study, we aimed to evaluate the effect of high-dose intravenous anakinra treatment on the development of thrombotic events in severe and critical COVID-19 patients.

## Materials and methods

### Patients and data

This retrospective observational study, which includes a secondary analysis of our previous study^13^, was conducted at a tertiary referral center in Aksaray, Turkey. The patients who had available data about thrombotic events both in two arms (anakinra and control group) from our previous study were re-evaluated and included for PS matching in this study. The patients with no data about thrombotic events and/or missing data were excluded. Diagnosis of COVID-19 was performed by typical computer tomography (CT) findings in addition to clinical signs and symptoms. All patients had positive polymerase chain reaction (PCR) and delta variant (B.1.617.2) proven by variant analysis.

The study population consisted of two groups as follows; the patients receiving high-dose intravenous anakinra (anakinra group) added to background therapy between 01.09.2021 and 01.02.2022 and the patients treated with standard of care (SoC) as historical control group who were hospitalized between 01.07.2021 and 01.09.2021. COVID-19 disease severity was evaluated according to the National Institute of Health (NIH) severity scale and only severe (NIH score 3; patients with SpO_2_ < 94% on room air at sea level, PaO_2_/FiO_2_ < 300 mm Hg, a respiratory rate > 30 breaths/min, or lung infiltrates > 50%) and critically ill (NIH score 4; patients have acute respiratory distress syndrome, septic shock that may represent virus-induced distributive shock, cardiac dysfunction, an exaggerated inflammatory response, and exacerbation of underlying comorbidities, as well as requirement of high flow nasal oxygen therapy [HFNO] or invasive mechanical ventilation [IMV]) patients in the ward were included into the study^14^.

The study has been performed in accordance with the Declaration of Helsinki and the Health Insurance Portability and Accountability Act (HIPAA) Privacy Rule. An informed written consent was obtained for the study. Institutional Review Board approval was also obtained from the Aksaray University Ethics Committee (date/number: 24.02.2022, 2022/04-09).

### Laboratory evaluation

Laboratory values such as hemogram, liver enzymes, troponin levels, C-reactive protein (CRP) (mg/dL), ferritin (pg/mL), d-dimer (pg/mL), lactate dehydrogenase (LDH) (U/L), procalcitonin (pg/dL) at the admission and consecutive days (procalcitonin was every other day but others were once in a day); the peak levels of CRP, ferritin, d-dimer and LDH levels were recorded. The inflammatory state of the patients was evaluated and derived based on the COVID hyperinflammatory syndrome score (cHIS) and it was calculated according to the combination of neutrophil and lymphocyte counts at the admission and the peak levels of CRP, ferritin, d-dimer, and LDH during to the follow-up^15^. The item of fever was removed in both arms due to its lower frequency (< %10) and inconsistent relevance in the diagnosis of COVID-19-associated hyperinflammation. Therefore, the maximum score of the new version of the cHIS score was 5 points (modified cHIS [mcHIS] score) was calculated in both groups^13^.

### Treatment protocol and outcome

All patients received background corticosteroid therapy with intravenous 80 mg/day methylprednisolone or dexamethasone 6 mg/day (or its equivalent)^16^ and enoxaparin 0.4 mg/day at the admission and continued consecutive days (SoC). Anakinra was added to the background treatment in the early phase of the disease in patients who did not respond to initial treatment for at least two days or concomitantly with steroids in patients with higher risk and/or critical illness at admission. The average starting dose of anakinra was 400 mg/day intravenously and increased gradually to a maximum of 1600 mg/day if necessary (10 mg/kg/day). Anakinra dose adjustment was performed by the same experienced physician in COVID-19 (MB) according to daily clinical (respiratory symptoms, degree of oxygen supply, presence of fever) and laboratory findings. Anakinra and steroid treatment were administered until the hyperinflammatory response of the patients disappeared or the discharge or development of death.

Diagnosis of PTE was confirmed by thorax CT-angiography in patients with prominent d-dimer increase despite a decrease in acute phase reactants (APR) such as CRP and ferritin and/or increase in need of oxygen therapy and respiratory distress despite the decrease in levels of APRs. Diagnosis of acute coronary syndrome (ACS) was made according to the definition of the European Society of Cardiology guideline^17^. Severe infection was defined as the development of opportunistic infection, need for intravenous antibiotics, sepsis, or requirement of intensive care unit (ICU) admission or development of death due to secondary infection.

### Statistical analysis

In our study, the 22.0 version (IBM, Armonk, NY, USA) of the SPSS (Statistical Package for the Social Sciences) program was used for statistical analysis of data. In descriptive statistics, discrete​ ​and continuous numerical variables were expressed as mean, ± standard deviation, or median (minimum–maximum), interquartile range (IQR). Categorical variables were expressed as number of cases (%). Cross-table statistics were used to compare categorical variables (Chi-Square, Fisher’s exact test). Normally distributed parametric data were compared with Student's t-test and non-parametric data that did not meet normal distribution were compared with Mann–Whitney U and Kruskal–Wallis tests. Correlation analysis was performed by Pearson or Spearman method according to normality distribution. Kaplan–Meier and log-rank methods were used for survival analysis. Multivariate analysis was performed by using logistic regression. Sensitivity and specificity calculations were performed by Receiver operating characteristic (ROC) analysis. p < 0.05 value was considered statistically significant.

### Propensity score matching

The first step in Propensity Score Matching (PSM) is to identify the covariates from which to calculate propensity scores (PS). Age, gender, mcHIS scores, and comorbidities such as DM, HT, and CHD of the patients were determined as the variables to be matched. The PS matching was done as 1:1 with the nearest neighbor method. The caliper value was 0.2. When matching, we performed this analysis by assigning values according to the averages of the parameters with missing data. PSM was performed with the SPSS package program 28.0.1 using the R package program and an auxiliary plugin (PS matching 3.0 SPE). Dot-plot of standardized mean differences for all covariates before and after PS matching was shown in Supplementary Fig. 1. Jitter plots for trend scores and line plots of standardized differences were described in supplemental Figs. 2 and 3, respectively.

### Ethics approval

The study has been performed in accordance with the Declaration of Helsinki and the Health Insurance Portability and Accountability Act (HIPAA) Privacy Rule. An informed written consent was obtained for the study. Institutional Review Board approval was also obtained from the Aksaray University Ethics Committee (date/number: 24.02.2022, 2022/04-09).

## Results

### Analysis before PS matching

We included 114 patients in SoC and 139 patients in the Anakinra group in the study. The baseline clinical and laboratory features of the patients are described in Table 1.

**Table 1:** Baseline clinical and laboratory features and outcomes of the patients before and after Propensity-score (PS) Matching.

Variables	Before PS matching			After PS matching		
	Anakinra (n = 139)	SoC (n = 114)	p value (OR)	Anakinra (n = 88)	SoC (n = 88)	p value (OR)
Age, years, median (IQR)	71 (25)	65.5 (23)	0.09	70 (29)	66.5 (24)	0.6
Gender, male, n (%)	72 (51.8)	45 (39.5)	**0.05 (3.8)**	40 (45.5)	41 (46.6)	0.9
Duration of hospitalization (days), median (IQR)	11 (12)	9 (7.3)	**0.03**	10 (13)	10 (9)	0.8
Comorbidities, n (%)						
Diabetes mellitus	36/137 (26.3)	39 (34.2)	0.17	27 (30.7)	29 (33)	0.7
Hypertension	79/135 (58.5)	64 (56)	0.7	49/86 (57)	53 (60.2)	0.7
Coronary heart disease	24/135 (17.8)	24 (21)	0.5	19/86 (22)	19 (21.6)	0.9
Chronic renal failure	28 (20)	6 (5.3)	**0.001 (11.9)**	15/75 (19.2)	22 (25)	0.4
Chronic obstructive lung disease	22/136 (16.2)	19 (16.7)	0.9	14/86 (16.3)	14 (16)	1
Malignancy	16/138 (11.6)	8 (7)	0.2	6 (6.8)	8 (9.1)	0.6
Vaccination history	44/86 (51.2)	26/63 (41.3)	0.2	31/58 (53.4)	18/48 (37.5)	0.1
Disease severity, n (%)						
NIH score 3 (severe)	54 (38.8)	68 (59.6)	**0.001 (10.9)**	36 (41)	46 (52)	0.1
NIH score 4 (Critical)	85 (61.2)	46 (40.4)		52 (59)	42 (48)	
Vaccination history, median (IQR)	2 (1)	2 (0)	0.9	3 (1)	2 (1.5)	0.13
mcHIS score, median (IQR)	3 (1)	3 (3)	** < 0.001**	3 (2)	3 (2)	0.5
Laboratory results						
Neutrophil to lymphocyte ratio, median (IQR)	6.8 (8)	4.4 (4.44)	**0.002**	6.9 (8.2)	4.6 (5.4)	0.06
Hemoglobin (g/L), mean ± SD	13.2 ± 2.2	13.2 ± 2	0.6	13.3 ± 2.3	13.2 ± 2	0.5
Creatinine (mg/dL), median (IQR)	0.9 (0.47)	0.83 (0.52)	0.5	0.84 (0.54)	0.9 (0.68)	0.4
Procalcitonin (pg/dL), median (IQR)	0.2 (0.46)	0.16 (0.31)	0.7	0.18 (0.43)	0.18 (0.62)	0.5
C-reactive protein (mg/L), median (IQR)						
1	116 (113)	100.3 (100.3)	0.4	115 (133)	107 (107)	0.9
2	148 (120)	126 (88)	**0.012**	141 (152)	141.2 (90)	0.7
3	11.4 (64)	13.1 (91)	0.5	10.4 (58.8)	14.5 (101)	0.2
Ferritin (pg/mL), median (IQR)						
1	393 (592)	322 (423)	0.076	334.5 (590.5)	302 (371)	0.16
2	714 (969)	378 (660)	** < 0.001**	630 (811)	495 (873)	0.12
3	392 (590)	268 (480)	**0.007**	379 (427)	313 (630)	0.7
d-dimer (pg/mL), median (IQR)						
1	1.2 (1.1)	0.85 (1.05)	**0.04**	1.24 (1.14)	1 (1)	**0.05**
2	4.1 (12.2)	2.25 (5)	**0.002**	2.75 (14.8)	2.7 (6.8)	0.4
3	1.4 (4.1)	1.14 (2.14)	0.15	1.37 (3.3)	1.2 (3.6)	0.9
Lactate dehydrogenase (U/L), median (IQR)						
1	404 (220)	414 (229)	0.7	398.5 (219)	399.5 (210)	0.5
2	559 (266)	408 (237)	** < 0.001**	570 (259)	425 (269)	** < 0.001**
3	357 (231)	334 (170)	**0.03**	366.5 (204)	345.5 (195)	0.15
Outcomes, n (%)						
Severe infection	19/128 (14.8)	26 (22.8)	0.1	13/82 (16)	25 (28.4)	**0.05 (3.9)**
Pneumothorax	3/134 (2.2)	0	0.1	2/86 (2.3)	0,00	0.15
Development of any thrombotic event	7 (5)	14 (12.3)	**0.038 (4.3)**	3 (3.4)	14 (15.9)	**0.005 (7.9)**
Pulmonary thromboembolism	4 (2.9)	11 (9.6)	**0.023 (5.1)**	3 (3.4)	11 (12.5)	**0.026 (5)**
Acute coronary syndrome	3 (2.2)	6 (5.3)	0.2	0	6 (6.8)	**0.013 (6.2)**
ICU requirement	55 (39.6)	25 (22)	**0.003 (9)**	33 (37.5)	24 (27.3)	0.2
Mortality	51 (36.7)	27 (23.7)	**0.026 (5)**	30 (34.1)	25 (28.4)	0.4

Significant values are in bold.

*PS* Propensity score, *SoC* Standard of care, *OR* Odds ratio, *IQR* Interquartile range, *ICU* Intensive care unit, *1* Baseline levels, *2* Peak levels, *3* Last levels.

Development of any thromboembolic event (5% vs 12.3%, p = 0.038; OR 4.3) and PTE (2.9% vs 9.6%, p = 0.023; OR 5.1) were lower in the Anakinra group than SoC. No patient experienced CVA and/or clinically evident DVT both in two arms. Although severe infection, pneumothorax, and ACS were not different between the two arms (p = 0.1, p = 0.1, and p = 0.2, respectively); ICU admission (39.6% vs 22%, p = 0.003; OR 9) and mortality (36.7% vs 27%, p = 0.026; OR) were higher in Anakinra group compared to SoC before PS matching analysis (Table 1).

Patients experienced any thromboembolic event had longer duration of hospitalization (p = 0.03), higher vaccination counts (p = 0.028), more frequent CHD (p = 0.001; OR 11.8), critical disease (p = 0.001; OR 10.6), higher mcHIS scores (p < 0.001), lower NLR (p = 0.002) and higher baseline d-dimer levels (p = 0.04), higher peak levels of CRP (p = 0.012), ferritin (p < 0.001), d-dimer (p = 0.002), and LDH (p < 0.001). Development of thrombosis was also higher in patients who had mortality (62% vs 28%, p = 0.001; OR 10.4) in univariate analysis (Table 2). Patients developed PTE had longer duration of hospitalization (p = 0.03), higher vaccination counts (p = 0.03), critical disease (p = 0.005; OR 7.8), higher mcHIS scores (p < 0.001), and higher baseline d-dimer levels (p = 0.04), higher peak levels of CRP (p = 0.012), ferritin (p < 0.001), d-dimer (p = 0.002), and LDH (p < 0.001). Development of PTE was also higher in patients who had a severe infection (p = 0.028; OR 4.8), pneumothorax (p = 0.046; OR 4), ACS (p < 0.001; OR 12.6), and SoC (p = 0.023; OR 5.1) in univariate analysis (Table 3). In multivariate analysis, peak d-dimer levels (p < 0.001, OR 1.1, 95% Confidence interval [CI] 1.05–1.16), critical illness (p = 0.044, OR 9.5, 95% CI 1.06–85.5), and SoC (compared to Anakinra) (p = 0.002, OR 11.2, 95% CI 2.47–51.1) were associated with development of any thromboembolic event (supplementary table).

**Table 2:** Univariate analysis of the patients who had any thromboembolic event before and after Propensity-score (PS) Matching.

Variables	Patients with thrombosis before PS matching			Patients with thrombosis after PS matching		
	Yes (n = 21)	No (n = 232)	p value (OR)	Yes (n = 17)	No (n = 159)	p value (OR)
Age, years, median (IQR)	71 (22)	68 (25)	0.09	71 (26)	69 (26)	0.5
Gender, male, n (%)	13 (62)	104(45)	0.1	10 (58.8)	71 (44.7)	0.3
Duration of hospitalization (days), median (IQR)	11 (10)	9.5 (10)	**0.03**	11 (10)	10 (10)	0.4
Comorbidities, n (%)						
Diabetes mellitus	7 (33.3)	68/230 (29.6)	0.7	5 (29.4)	51 (32)	0.8
Hypertension	13 (62)	130/228 (57)	0.7	10 (58.8)	92 (58.6)	1
Coronary heart disease	10 (47.6)	38/228 (16.7)	**0.001 (11.8)**	7 (41.2)	31 (19.7)	**0.04 (4.1)**
Chronic renal failure	4 (19)	30 (13)	0.4	4 (23.5)	33 (22)	0.9
Chronic obstructive lung disease	4 (19)	37/229 (16.2)	0.7	4 (23.5)	24 (15.3)	0.4
Malignancy	3 (14.3)	21/231 (9)	0.4	3 (17.6)	11 (7)	0.12
Vaccination history	5/13 (38.5)	65/136 (48)	0.5	4/10 (40)	45/96 (47)	0.7
Disease severity, n (%)						
NIH score 3 (severe)	3 (14.3)	119 (51.3)	**0.001 (10.6)**	1 (6)	81 (61)	** < 0.001 (12.5)**
NIH score 4 (Critical)	18 (85.7)	113 (48.7)		16 (94)	78 (49)	
Vaccination counts, median (IQR)	3 (1.5)	2 (1)	**0.028**	2.5 (1.75)	2 (1)	1
mcHIS score, median (IQR)	4 (2)	3 (2)	** < 0.001**	4 (2)	3 (2)	**0.001**
Laboratory results						
Neutrophil to lymphocyte ratio, median (IQR)	5.6 (10.5)	5.6 (5.8)	**0.002**	4 (10.8)	5.9 (6.7)	0.7
Hemoglobin (g/L), mean ± SD	12.6 ± 1.7	13.3 ± 2.2	0.6	12.4 ± 1.5	13.3 ± 2.2	**0.03**
Creatinine (mg/dL), median (IQR)	0.94 (0.73)	0.84 (0.48)	0.5	0.94 (0.67)	0.85 (0.53)	0.7
Procalcitonin (pg/dL), median (IQR)	0.2 (0.7)	0.2 (0.43)	0.7	0.12 (1.1)	0.2 (0.45)	
C-reactive protein (mg/L), median (IQR)						
1	118 (123)	108 (107)	0.4	110 (106)	107 (119)	0.9
2	212.5 (121)	137.5 (95)	**0.012**	212.5 (113)	135 (98)	** < 0.001**
3	87.4 (144)	11.5 (64)	0.5	121 (152)	11.4 (75)	**0.014**
Ferritin (pg/mL), median (IQR)						
1	204.5 (603)	371 (545)	0.08	172 (223)	336 (544)	**0.04**
2	714 (735)	546 (867)	** < 0.001**	694 (735)	532 (853)	0.4
3	551.4 (695)	331.5 (483)	**0.007**	551 (680)	331.5 (483)	**0.044**
d-dimer (pg/mL), median (IQR)						
1	1.44 (2)	1.15 (1.1)	**0.04**	0.75 (1.95)	1.2 (1.1)	0.4
2	21 (28)	2.7 (7.3)	**0.002**	23.8 (25)	2.56 (6.2)	** < 0.001**
3	5.6 (32.7)	1.2 (2.3)	0.15	19.7 (32)	1.18 (1.9)	** < 0.001**
Lactate dehydrogenase (U/L), median (IQR)						
1	418 (268)	409 (215)	0.7	418 (154)	398 (207)	0.8
2	655 (487)	476 (271)	** < 0.001**	663 (540)	490 (277)	**0.038**
3	482 (518)	348 (169)	**0.03**	482 (506)	351 (164)	**0.012**
Outcomes, n (%)						
Severe infection	7 (33.3)	38/221 (17.2)	0.07	7 (41)	31/153 (20.3)	**0.05 (3.9)**
Pneumothorax	1 (4.8)	2/227 (0.9)	0.1	1 (6)	1/157 (0.6)	0.054
Treatment						
Anakinra	7 (5)	132 (95)	**0.038 (4.3)**	3 (17.6)	85 (53.5)	**0.005 (7.9)**
SoC	14 (12.3)	100 (87.7)		14 (82.4)	74 (46.5)	
ICU requirement	10 (47.6)	70 (30.2)	0.1	8 (47)	49 (31)	0.17
Mortality	13 (62)	65 (28)	**0.001 (10.4)**	11 (64.7)	44 (27.7)	**0.002 (9.8)**

Significant values are in bold.

*PS* Propensity score, *SoC* Standard of care, *OR* Odds ratio, *IQR* Interquartile range, *ICU* Intensive care unit, *1* Baseline levels, *2* Peak levels, *3* Last levels.

**Table 3:** Univariate analysis of the patients who had pulmonary thromboembolism before and after Propensity-score (PS) Matching.

Variables	Patients with pulmonary thromboembolism before PS matching			Patients with pulmonary thromboembolism after PS matching		
	Yes (n = 15)	No (n = 238)	p value (OR)	Yes (n = 14)	No (n = 162)	p value (OR)
Age, years, median (IQR)	71 (23)	68.5 (25)	0.09	68.5 (22)	69.5 (27)	0.9
Gender, male, n (%)	8 (53.3)	109 (45.8)	0.6	8 (57)	73 (45)	0.4
Duration of hospitalization (days), median (IQR)	10 (5)	10 (10)	**0.03**	10.5 (5.75)	10 (10)	0.6
Comorbidities, n (%)						
Diabetes mellitus	3 (20)	72/236 (30.5)	0.4	3 (21.4)	53 (32.7)	0.4
Hypertension	8 (53.3)	135/234 (57.7)	0.7	8 (57)	94/160 (58.8)	0.9
Coronary heart disease	5 (33.3)	43/234 (18.4)	0.16	5 (35.7)	33/160 (20.6)	0.2
Chronic renal failure	3 (20)	31 (13)	0.4	3 (21.4)	21 (13)	0.4
Chronic obstructive lung disease	3 (20)	38/235 (16.2)	0.7	3 (21.4)	25/160 (15.6)	0.6
Malignancy	2 (13.3)	22/237 (9.3)	0.6	2 (14.3)	12 (7.4)	0.4
Vaccination history	4/10 (40)	66/139 (47.5)	0.6	4/9 (44.4)	45/97 (46.4)	0.9
Disease severity, n (%)						
NIH score 3 (severe)	2 (13.3)	120 (50.4)	**0.005 (7.8)**	1 (7)	81 (50)	**0.002 (9.5)**
NIH score 4 (Critical)	13 (86.7)	118 (49.6)		13 (93)	81 (50)	
Vaccination history, median (IQR)	2.5 (1.75)	2 (1)	**0.03**	2.5 (1.75)	2 (1)	1
mcHIS score, median (IQR)	4 (2)	3 (2)	** < 0.001**	4.5 (2)	3 (2)	**0.002**
Laboratory results						
Neutrophil to lymphocyte ratio, median (IQR)	7.6 (12.2)	5.6 (5.7)	0.5	7.5 (12)	5.8 (6.5)	0.8
Hemoglobin (g/L), mean ± SD	12.6 ± 1.9	13.3 ± 2.1	0.6	12.2 ± 1.5	13.6 ± 2.2	**0.02**
Creatinine (mg/dL), median (IQR)	0.94 (0.7)	0.84 (0.5)	0.5	1 (0.7)	0.85 (0.52)	0.6
Procalcitonin (pg/dL), median (IQR)	0.13 (0.5)	0.2 (0.43)	0.7	0.1 (1.1)	0.2 (0.45)	0.7
C-reactive protein (mg/L), median (IQR)						
1	110 (110)	108 (105)	0.4	114 (120)	107 (118)	0.6
2	212.5 (122)	138.6 (96)	**0.012**	216 (119)	136.5 (97.4)	** < 0.001**
3	87.4 (158)	11.6 (68)	0.5	91 (160)	11.6 (80.5)	0.1
Ferritin (pg/mL), median (IQR)						
1	203 (413)	379 (543.5)	0.08	172.4 (223)	336 (544)	**0.04**
2	693.6 (739)	552 (863)	** < 0.001**	633 (800)	545.7 (853)	0.6
3	551.4 (614)	335.5 (487)	**0.007**	533.7 (699)	333 (486)	0.18
d-dimer (pg/mL), median (IQR)						
1	1.1 (4.8)	1.17 (1.1)	**0.04**	0.75 (1.95)	1.2 (1.1)	0.4
2	31.8 (18.2)	2.7 (7.4)	**0.002**	33.4 (18.6)	2.6 (6.3)	** < 0.001**
3	22.3 (31.5)	1.2 (2.3)	0.15	27 (31)	1.2 (1.9)	** < 0.001**
Lactate dehydrogenase (U/L), median (IQR)						
1	428.5 (207)	409 (219)	0.7	418 (154)	398 (207)	0.8
2	633 (426)	477 (282)	** < 0.001**	615.5 (468)	496 (282)	0.16
3	482 (495)	348 (171)	**0.03**	472 (517)	355 (166)	**0.044**
Outcomes, n (%)						
Severe infection	6 (40)	39/227 (17.2)	**0.028 (4.8)**	6 (43)	32/156 (20.5)	0.055
Pneumothorax	1 (6.7)	2/233 (0.9)	**0.046 (4)**	1 (7)	1/160 (0.6)	**0.03 (4.8)**
Acute coronary syndrome	3 (20)	6 (2.5)	** < 0.001 (12.6)**	3 (21.4)	3 (1.9)	** < 0.001 (15)**
Treatment						
Anakinra	4 (3)	135 (97)	**0.023 (5.1)**	3 (3.4)	85 (96.6)	**0.026 (5)**
SoC	11 (9.6)	103 (90.4)		11 (12.5)	77 (87.5)	
ICU requirement	5 (33.3)	75 (31.5)	0.9	5 (35.7)	52 (32)	0.8
Mortality	8 (53.3)	70 (29.4)	0.052	8 (57)	47 (29)	**0.03 (4.7)**

Significant values are in bold.

*PS* Propensity score, *SoC* Standard of care, *OR* Odds ratio, *IQR* Interquartile range, *ICU* Intensive care unit, *1* Baseline levels, *2* Peak levels, *3* Last levels.

### Analysis after PS matching

After 1:1 PS matching, 88 patients in SoC and 88 patients in the Anakinra group were matched and included in the analysis. The baseline clinical and laboratory features of the patients are described in Table 1. Severe infection (28.4% vs 16%, p = 0.05; OR 3.9), development of any thromboembolic event (15.9% vs 3.4%, p = 0.005; OR 7.9), PTE (12.5% vs 3.4%, p = 0.026; OR 5), ACS (6.8% vs 0, p = 0.013; OR 6.2) were higher in SoC arm compared to Anakinra.

Patients who experienced any thromboembolic event had more frequent CHD (p = 0.04; OR 4.1), critical illness (p < 0.001; OR 12.5), lower hemoglobin and baseline ferritin levels (p = 0.03 and p = 0.04, respectively), higher mcHIS scores (p = 0.001), higher peak levels of CRP (p < 0.001), d-dimer (p < 0.001), LDH (p = 0.038). Furthermore, severe infection (41% vs 20.3%, p = 0.05; OR 3.9) and mortality (64.7% vs 27.7%, p = 0.002; OR 9.8) were higher in patients who had any thromboembolic event than those had not (Table 2). Similarly, PTE was higher in patients who had a critical illness (p = 0.002; OR 9.5), lower hemoglobin and ferritin levels (p = 0.02 and p = 0.04, respectively), higher mcHIS scores (p = 0.002), peak levels of CRP (p < 0.001), d-dimer (p < 0.001), pneumothorax (p = 0.03; OR 4.8), ACS (p < 0.001; OR 15), and mortality (p = 0.03; OR 4.7) (Table 3). PTE development was associated with peak levels of d-dimer levels (p = 0.02, OR 1.08, 95% CI 1.01–1.15) in multivariate analysis.

Development of ACS was higher in patients who had a history of CHD and malignancy (p = 0.007; OR 7.3 and p = 0.02; OR 5.5, respectively), critical illness (p = 0.02; OR 5.4), higher mcHIS scores (p = 0.02), peak levels of CRP (p = 0.043), d-dimer (p = 0.03), LDH (p = 0.004) (Table 4). ACS was also higher in SoC (P = 0.016; OR 6.2) and patients had mortality (p < 0.001; OR 13.7) in univariate analysis. Development of ACS was associated with the history of CHD (p = 0.038, OR 6.9, 95% CI 1.1–42.3) and PTE (p = 0.008, OR 11.5, 95% CI 1.9–69.5) in multivariate analysis.

**Table 4:** Univariate analysis of the patients who had acute coronary syndrome after propensity-score (PS) matching.

Variables	Patients with ACS after PS matching		
	Yes (n = 6)	No (n = 170)	p value (OR)
Age, years, median (IQR)	77.5 (33)	69 (26)	0.1
Gender, male, n (%)	5 (83.3)	76 (44.7)	0.06
Duration of hospitalization (days), median (IQR)	9 (14.5)	10 (9.3)	0.9
Comorbidities, n (%)			
Diabetes mellitus	2 (33.3)	54 (31.8)	0.9
Hypertension	3 (50)	99/168 (59)	0.7
Coronary heart disease	4 (66.7)	34/168 (20.2)	**0.007 (7.3)**
Chronic renal failure	1 (16.7)	23 (13.5)	0.8
Chronic obstructive lung disease	2 (33.3)	26/168 (15.5)	0.2
Malignancy	2 (33.3)	12 (7)	**0.02 (5.5)**
Vaccination history	1/4 (25)	48/102 (47)	0.4
Disease severity, n (%)			
NIH score 3 (severe)	0	82 (48.2)	**0.02 (5.4)**
NIH score 4 (Critical)	6 (100)	88 (51.8)	
Vaccination history, median (IQR)		2 (1)	0.5
mcHIS score, median (IQR)	4.5 (1.25)	3 (2)	**0.02**
Laboratory results			
Neutrophil to lymphocyte ratio, median (IQR)	4 (6)	5.9 (6.8)	0.6
Hemoglobin (g/L), mean ± SD	12.8 ± 1.3	13.3 ± 2.2	0.5
Creatinine (mg/dL), median (IQR)	1 (0.66)	0.87 (0.54)	0.5
Procalcitonin (pg/dL), median (IQR)	NA	0.18 (0.44)	NA
C-reactive protein (mg/L), median (IQR)			
1	129 (164)	107 (117)	0.6
2	144.8 (74)	138.6 (110)	**0.043**
3	207.6 (80)	11.5 (80)	**0.003**
Ferritin (pg/mL), median (IQR)			
1	NA	331 (545)	NA
2	1001 (761)	542 (848)	0.096
3	1001 (687)	333 (483)	**0.009**
d-dimer (pg/mL), median (IQR)			
1	NA	1.2 (1.1)	NA
2	27.3 (32.1)	2.7 (8.6)	**0.03**
3	27.3 (33)	1.2 (2.8)	**0.005**
Lactate dehydrogenase (U/L), median (IQR)			
1	390 (97)	399 (206)	0.8
2	998 (759)	488 (278)	**0.004**
3	582.5 (684)	355 (172)	**0.016**
Outcomes, n (%)			
Severe infection	3 (50)	35/164 (21.3)	0.1
Pneumothorax	0	2/168 (1.2)	0.8
Treatment			
Anakinra	0	88 (100)	**0.013 (6.2)**
SoC	6 (6.8)	82 (93.2)	
ICU requirement	4 (66.7)	53 (31.2)	0.07
Mortality	6 (100)	49 (28.8)	** < 0.001 (13.7)**

Significant values are in bold.

*NA: Not  available, PS* Propensity score, *SoC* Standard of care, *OR* Odds ratio, *IQR* Interquartile range, *ICU* Intensive care unit, *ACS* Acute coronary syndrome, *1* Baseline levels, *2* Peak levels, *3* Last levels.

**Figure 1:**
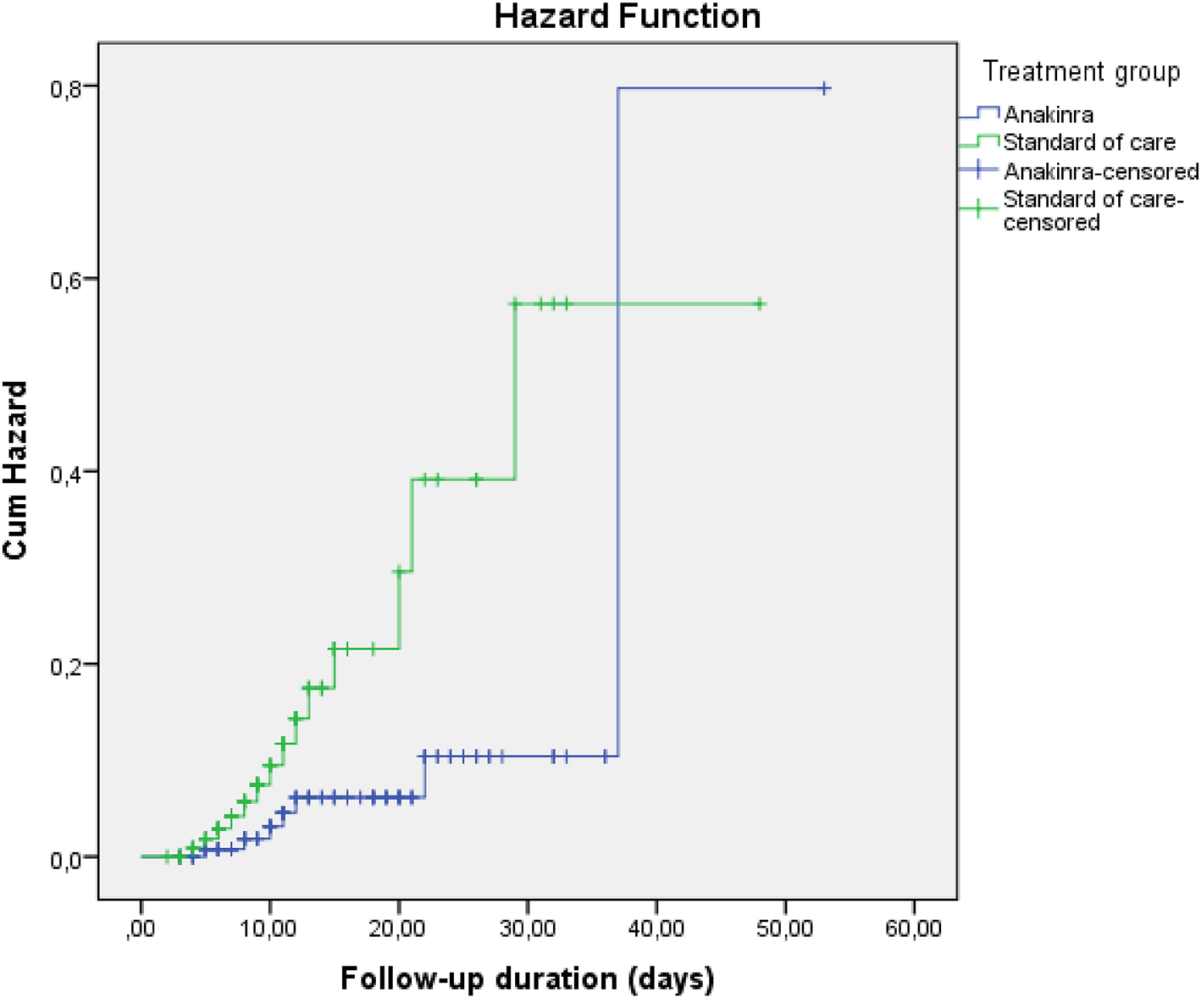
Development of any thromboembolic event in patients with COVID-19 according to the treatment groups (Kaplan–Meier survival analysis). Log-Rank; p = 0.003.

**Figure 2:**
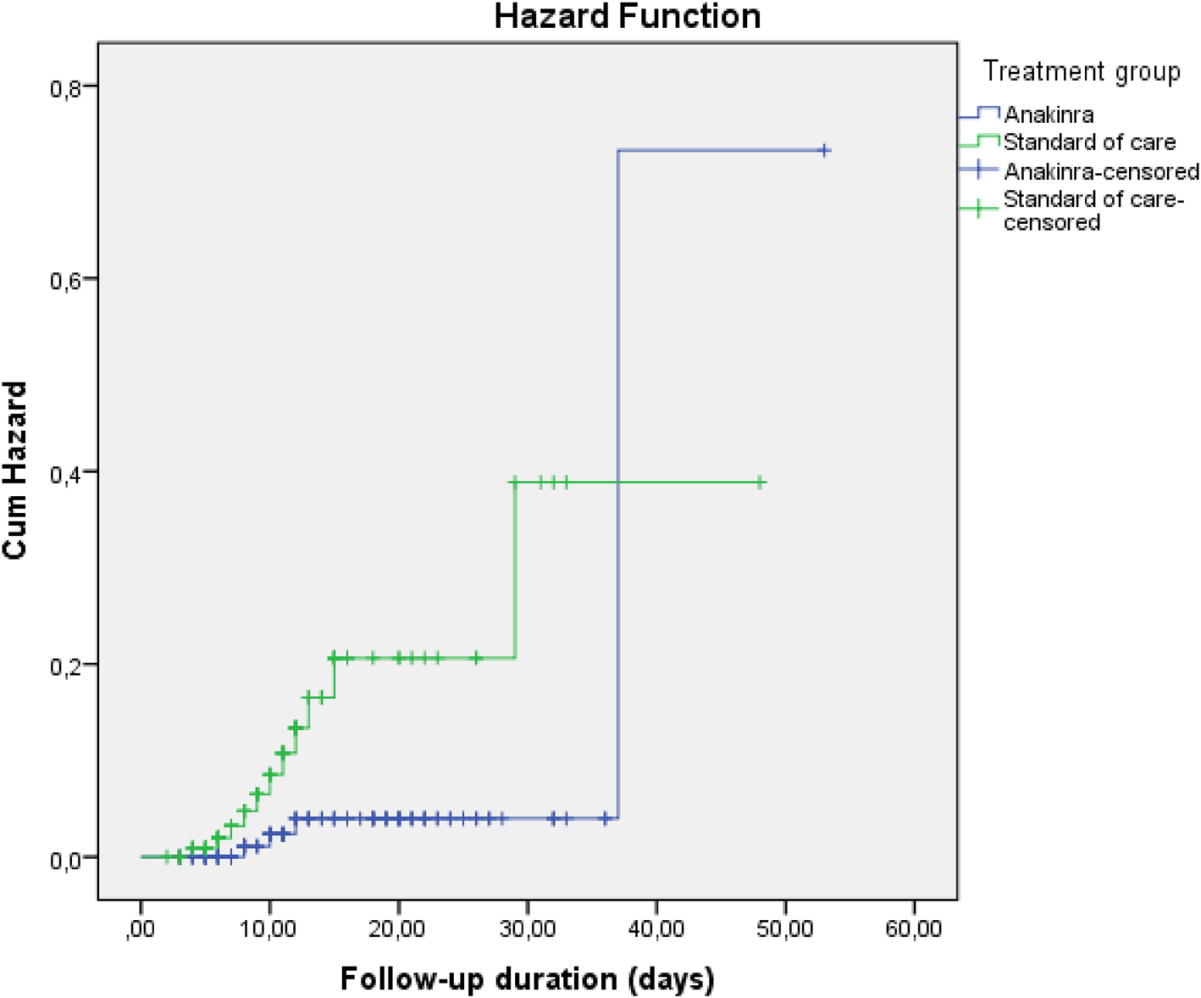
Development of pulmonary thromboembolism in patients with COVID-19 according to the treatment groups (Kaplan–Meier survival analysis). Log-Rank; p = 0.003.

**Figure 3:**
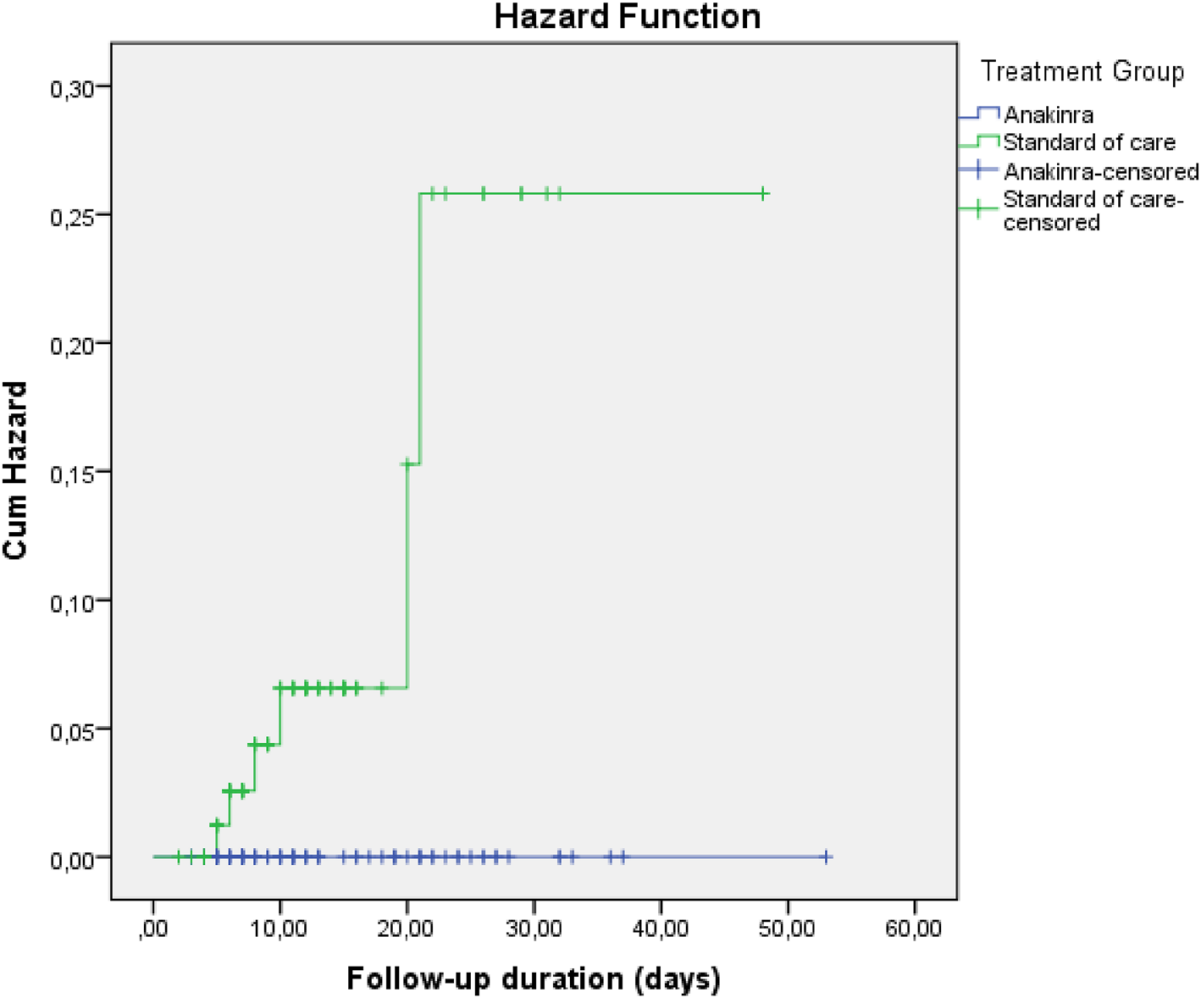
Development of acute coronary syndrome in patients with COVID-19 according to the treatment groups (Kaplan–Meier survival analysis). Log-Rank; p = 0.007.

**Figure 4:**
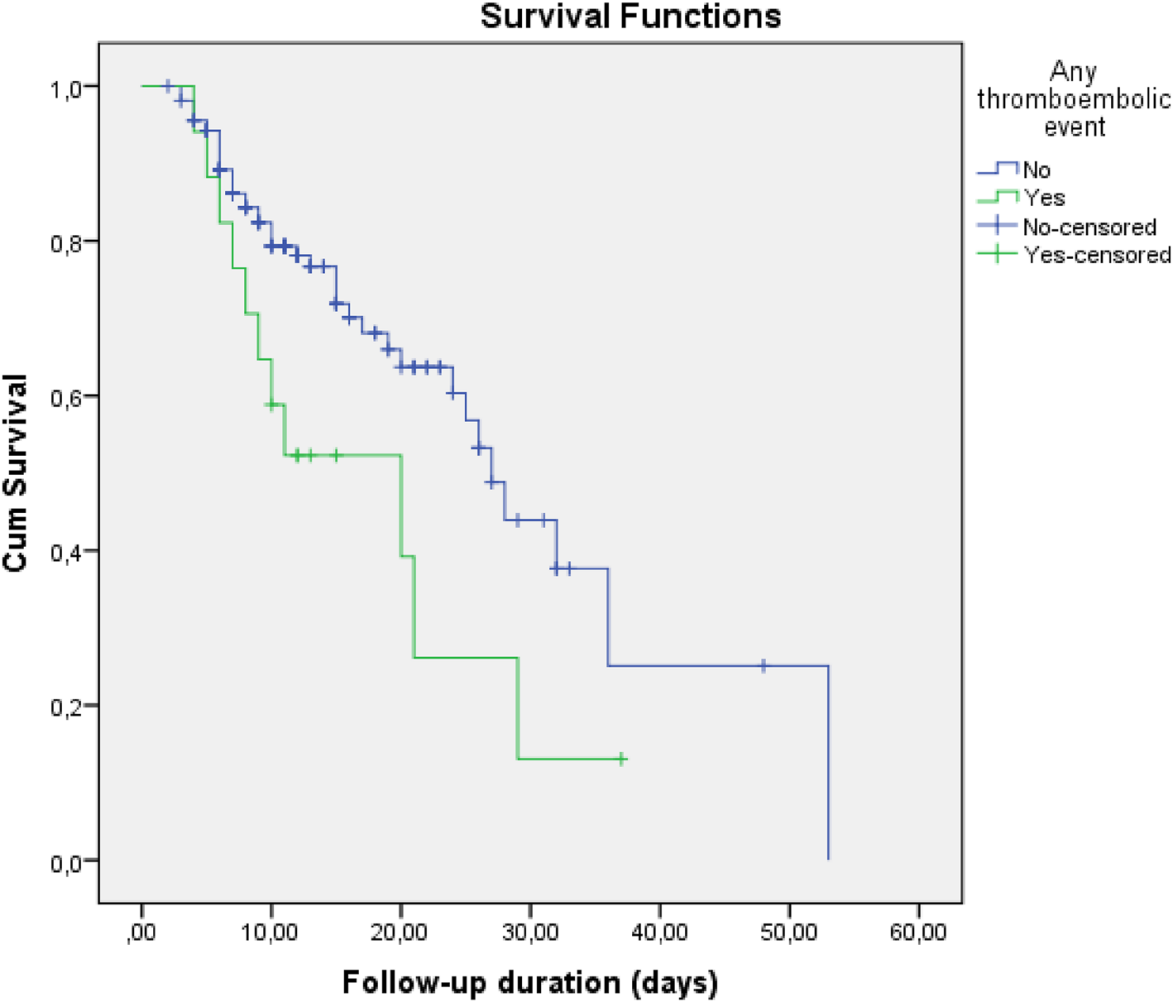
The survival rate of patients with COVID-19 according to the presence of any thromboembolic event (Kaplan–Meier survival analysis). Log-Rank; p = 0.03.

**Figure 5:**
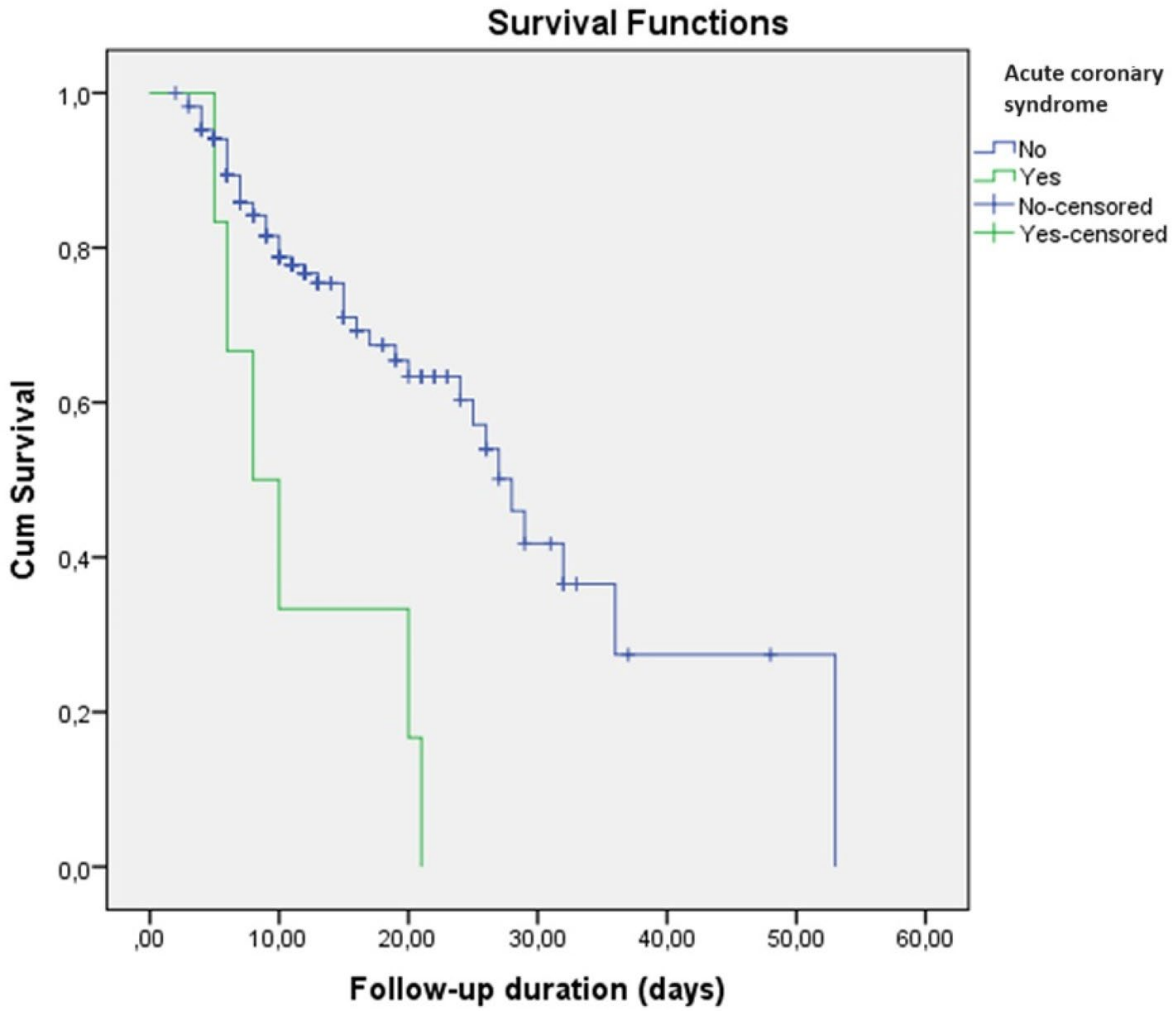
The survival rate of patients with COVID-19 (in the whole study group) according to the presence of acute coronary syndrome (Kaplan–Meier survival analysis). Log-Rank; p < 0.001.

In survival analysis, development of any thromboembolic event, PTE, and ACS were higher in SoC compared to Anakinra [Log-Rank; p = 0.003 (Fig. 1), p = 0.003 (Fig. 2), and p = 0.007 (Fig. 3), respectively]. Survival rate was also lower in patients with the SoC arm than Anakinra in patients who had any thromboembolic event as well as ACS [Log-Rank; p = 0.03 (Fig. 4) and p < 0.001 (Fig. 5), respectively]. The survival rate of patients with and without PTE did not differ in patients with COVID-19 (Supplementary Fig. 4).

ROC analysis revealed a cut-off value of d-dimer for the development of any thromboembolic event 16.75 (Area under curve [AUC] 0.804, p < 0.001 [95% CI 0.710–0.898]), for the development of PTE 14.97 (AUC 0.867, p < 0.001 [95% CI 0.774–0.960]), for the development of ACS 5.83 (AUC 0.736, p < 0.016 [95% CI 0.585–0.887]) (Supplementary Figs. 5, 6, and 7, respectively). Cut-off value of mcHIS score for the development of any thromboembolic event 3.5 (AUC 0.726, p = 0.001 [95% CI 0.632–0.821]), for the development of PTE 3.5 (AUC 0.740, p = 0.002 [95% CI 0.624–0.855]), for the development of ACS 3.5 (AUC 0.750, p = 0.01 [95% CI 0.630–0.870]) (Supplementary Fig. 8, 9, and 10, respectively). A cut-off value of peak levels of CRP for the development of any thromboembolic event 171.2 mg/L (AUC 0.780, p < 0.001 [95% CI 0.684–0.875]), for the development of PTE 201 mg/L (AUC 0.800, p < 0.001 [95% CI 0.694–0.905]), for the development of ACS 145.3 mg/L (AUC 0.743, p = 0.043 [95% CI 0.629–0.857]) (Supplementary Figs. 11, 12, and 13, respectively). Other results of ROC analysis are shown in Table 5 and Supplementary Figs. 14 and 15.

**Table 5:** ROC analysis of laboratory features of the patients for development of thromboembolic events in patients with COVID-19.

Variables	Cut-off value	Area under curve	p value (95% CI)	Sensitivity	Specificity	Likelihood ratio
mcHIS score						
Any thrombosis	3.5	0.726	0.001 (0.632–0.821)	71.4	63.2	1.94
PTE	3.5	0.740	0.002 (0.624–0.855)	73.3	62.4	1.95
ACS	3.5	0.750	0.01 (0.630–0.870)	77.8	61.7	2
d-dimer (pg/mL)^a^						
Any thrombosis	16.75	0.804	< 0.001 (0.710–0.898)	61.9	84.8	4
PTE	14.97	0.867	< 0.001 (0.774–0.960)	86.7	83.5	5.3
ACS	5.83	0.736	0.016 (0.585–0.887)	66.7	66.7	2
C-reactive protein (mg/L)^a^						
Any thrombosis	171.2	0.780	< 0.001 (0.684–0.875)	76.5	72.3	2.8
PTE	201	0.800	< 0.001 (0.694–0.905)	71.4	78.4	3.3
ACS	145.3	0.743	0.043 (0.629–0.857)	100	54.7	2.2
Lactate dehydrogenase (U/L)^a^						
Any thrombosis	496	0.676	0.008 (0.551–0.801)	66.7	53.4	1.4
PTE	NS	NS	NS	NS	NS	NS
ACS	649.5	0.802	0.002 (0.675–0.928)	77.8	75.5	3.2
Ferritin (pg/mL)^a^						
Any thrombosis	NS	NS	NS	NS	NS	NS
PTE	NS	NS	NS	NS	NS	NS
ACS	NS	NS	NS	NS	NS	NS
Neutrophil–lymphocyte ratio						
Any thrombosis	NS	NS	NS	NS	NS	NS
PTE	NS	NS	NS	NS	NS	NS
ACS	NS	NS	NS	NS	NS	NS

^a^Peak levels of value, *PTE* Pulmonary thromboembolism, *ACS* Acute coronary syndrome, *CI* Confidence interval, NS: Not significant.

## Discussion

It is well known that higher mortality rates and poor outcomes are mainly associated with the development of cytokine storms in patients with COVID-19^18^. Cytokine storm is a hyperinflammatory state that is seen in several conditions such as hematological malignancies, infectious diseases, and rheumatological conditions including adult-onset still disease (AOSD), and systemic lupus erythematosus^19^. Development of cytokine storm depends on the excessive production of several cytokines including interleukin-1 (IL-1), IL-6, tumor necrosis factor-alpha (TNF-α), and type 1 interferon (IFN) triggered by SARS-CoV-2 in COVID-19^20^. Recent studies revealed the importance of pulmonary macrophages’ activation secondary to SARS-CoV-2 (23), which results in inflammasome activation in COVID-19^21,22^. Inflammasomes are essential in the host defense against microorganisms including viruses that are present in various innate immune cells such as neutrophils, macrophages, and dendritic cells. Activation of inflammasomes leads to the cleavage of pro-IL-1β to produce active IL-1β^23^.

Anakinra is an IL-1 receptor antagonist which is widely used in several rheumatological diseases such as FMF, AOSD, and gout^24–26^ and also several hyperinflammatory conditions such as cancer-related hemophagocytic syndrome, chimeric antigen receptor-modified (CAR) T cell-associated cytokine storm, and macrophage activation syndrome^27–29^. Safety and efficacy of Anakinra was also established in COVID-19-associated cytokine storm^3^. Intravenous and high-dose anakinra is an emerging therapeutic option both in rheumatology practice, hyperinflammatory conditions, and COVID-19^30–32^. Although a study revealed a lack of efficacy with intravenous anakinra in patients with Multisystem Inflammatory Syndrome in Children (MIS-C), the anakinra dose was lower (4 mg/kg) in this study compared to our study^33^. Additionally, MIS-C has different pathogenetic mechanisms from COVID-19-associated cytokine storm which adaptive immune system activation is more prominent in the former one. Furthermore, the concomitant use of intravenous immunoglobulin as a background therapy may have influenced the outcome of the former study. Intravenous administration of anakinra ensures higher and faster maximum plasma concentration compared to the subcutaneous form^34^. Daily dose adjustment of anakinra may allow early intervention of the cytokine storm according to daily clinical status, as well as withdrawing the drug in case of infection or other complications. Additionally, intravenous high-dose anakinra treatment reduced mortality in our previous study^13^. Although the mortality rate was higher in the anakinra group compared to SoC before PS matching, it was thought to be related to a higher proportion of the patients with severe/critical disease in the anakinra group than in SoC. After adjusting confounder factors such as age, gender, disease severity, laboratory parameters (CRP, ferritin, d-dimer, LDH, NLR), and comorbidities with PS matching, the mortality rate tended to be lower in the anakinra group compared to the SoC. PS matching aims to ensure homogeneity and comparability between groups which includes potential bias due to the nature of a retrospective study.

Thromboembolic events are common in COVID-19 which is a remarkable finding from the beginning of the pandemic^7^. In the Middeldorp et al. study overall VTE frequency was 20% which was higher in patients in the ICU (47%) than ward (3.3%). In the former study, ICU admission, increased d-dimer, and NLR levels were associated with the development of VTE which were similar to our results. Furthermore, a study also revealed an association between the development of thrombosis and a prior history of CHD, critical disease, and increased d-dimer levels which were consistent with our results^35^. Moreover, the fact that higher values of peak levels of CRP, d-dimer, LDH, and ferritin than those baseline levels emphasize the crucial role of hyperinflammation in the development of thrombotic events in our study. COVID-19 patients have higher levels of d-dimer due to inflammation without an evident thrombosis, so reference values are not adequate and do not help in the diagnosis of thrombotic events in patients with COVID-19^7^. Therefore establishing a cut-off of d-dimer for indicating thrombosis was important in patients with COVID-19 independent of the effect of COVID-19 itself.

In our study, the lower frequency of PTE in the anakinra group was a remarkable finding even though the anakinra group had more severe disease before PS matching. This finding persists after the PS matching procedure. As already known, endothelial dysfunction, thrombophilia, and stasis are the main contributors to the development of venous thrombosis according to Virchow’s triad. In COVID-19, endothelial dysfunction appears to be a more prominent factor in the development of thrombosis^36^. In our study, none of the patients with PTE had clinically evident DVT which suggests COVID-19-related pulmonary thrombosis is an in-situ thrombosis rather than embolism which was claimed by Gabrielli et. al. study^37^. In our study, all patients received background anticoagulant prophylaxis in two arms but could not prevent thrombotic events. This situation is recently defined as ‘inflammothrombosis’ which is similar to Behçet’s disease (BD) associated with venous thrombosis^36,38^. While DVT and PT (in situ thrombosis, not embolism) may develop in BD separately, DVT is not expected to cause embolism due to its inflammatory nature (firmly attached to the vascular wall). Therefore, the definition of pulmonary thrombosis may be more accurate than pulmonary embolism in patients with COVID-19 similar to BD. Furthermore, while anticoagulant therapy does not prevent vascular thrombosis in BD patients, anti-inflammatory treatment improves vascular outcomes such as recanalization and prevention of relapses^39^. However, it should be kept in mind that limited data is showing the efficacy of anti-inflammatory therapy as an anticoagulant effect in patients with COVID-19.

Inflammation is an important contributor to the development of cardiovascular events including ACS. During the pandemic arterial thrombotic events such as CVA and MI were increased in patients with COVID-19^40,41^. The NLRP3 (NOD [nucleotide oligomerization domain]-, LRR [leucine-rich repeat]-, and PYD [pyrin domain]-containing protein 3) [NLRP3] inflammasome, an innate immune signaling complex, is the key mediator of IL-1 family cytokine production. Recent evidence has shown that NLRP3 inflammasome activation has a crucial role in leading to higher IL-1 production for the development of ACS^42^. Furthermore, colchicine, an inflammasome inhibitor was found to be effective in the prevention of MI in patients with a history of ACS^43^. Similarly, canakinumab is an IL-1β monoclonal antibody that decreases composite cardiovascular events including MI, stroke, coronary revascularization, and cardiovascular death in the CANTOS study^44^. In our study, the decreased incidence of ACS with Anakinra was consistent with previous studies. Additionally, higher mcHIS scores in patients who had ACS compared with had not emphasized the crucial role of hyperinflammation in the development of arterial events similar to venous events.

This study has some strengths and limitations. The retrospective design of the study was the main limitation although the controlled design of the study adjusting potential confounders by PS matching was important to prevent bias. We could not perform Doppler USG screening in patients who had PTE since it did not cause a change in treatment and critical situation of the patients. Diagnosis of ACS could not be confirmed with cardiac catheterization due to the clinical status of extremely ill patients. Having missing data is also a limitation of the study. On the other hand, the fact that the study is conducted in a single center enables homogeneity in terms of patient population and treatment decisions that are made by a single physician.

## Conclusions

Thromboembolic events were seen despite the anticoagulant prophylaxis in our study. The development of thrombosis was associated with hyperinflammation in patients with severe and critical COVID-19. Intravenous high-dose anakinra treatment decreases both venous and arterial events in patients with severe and critical COVID-19.

### Supplementary Information

Supplementary Information.

## Data Availability

The dataset of the study is available from the corresponding author upon reasonable request.
