# Supplementary material for: Intravenous high-dose anakinra drops venous thrombosis and acute coronary syndrome in severe and critical COVID-19 patients: a propensity score matched study
Source: Sci Rep. 2024 May 29;14:12369. doi: 10.1038/s41598-024-62079-y (PMC11137068; doi:10.1038/s41598-024-62079-y)

**Supplementary table and figures**

**Supplementary table**: Multivariate analysis of factors associated with the development of thrombotic events (overall) in patients with COVID-19

| **Varibles** | **B** | **p value** | **OR** | **95% CI** |
| --- | --- | --- | --- | --- |
| Peak D-dimer levels | 0.096 | <0.001 | 1.1 | 1.05-1.16 |
| NIH COVID score 4 (compared to 3) | 2.254 | 0.044 | 9.5 | 1.06-85.5 |
| SoC (compared to Anakinra) | 2.419 | 0.002 | 11.2 | 2.47-51.1 |

**Supplementary figure 1**: Dot-plot of standardized mean differences for all covariates before and after PS matching


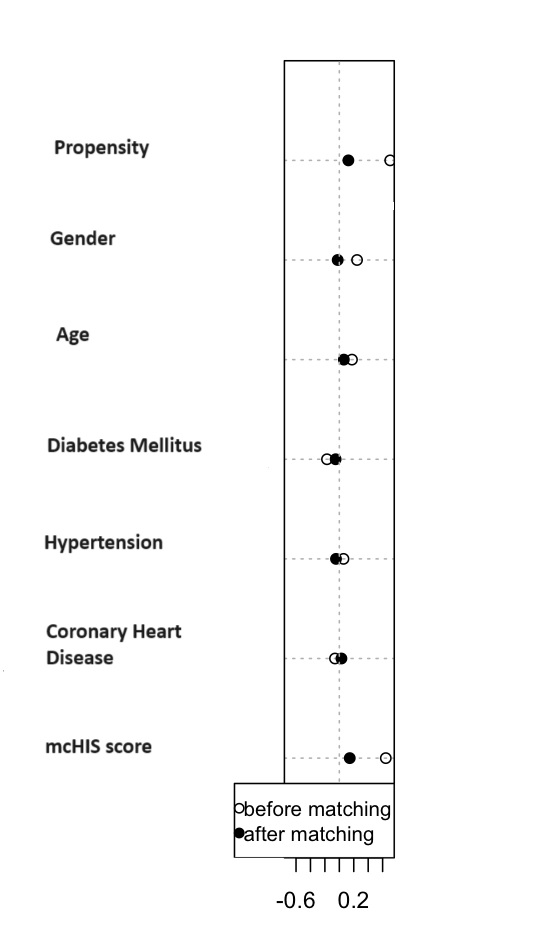


**Supplementary figure 2**: Jitter plots for trend scores by propensity score-matching analysis in patients with COVID-19


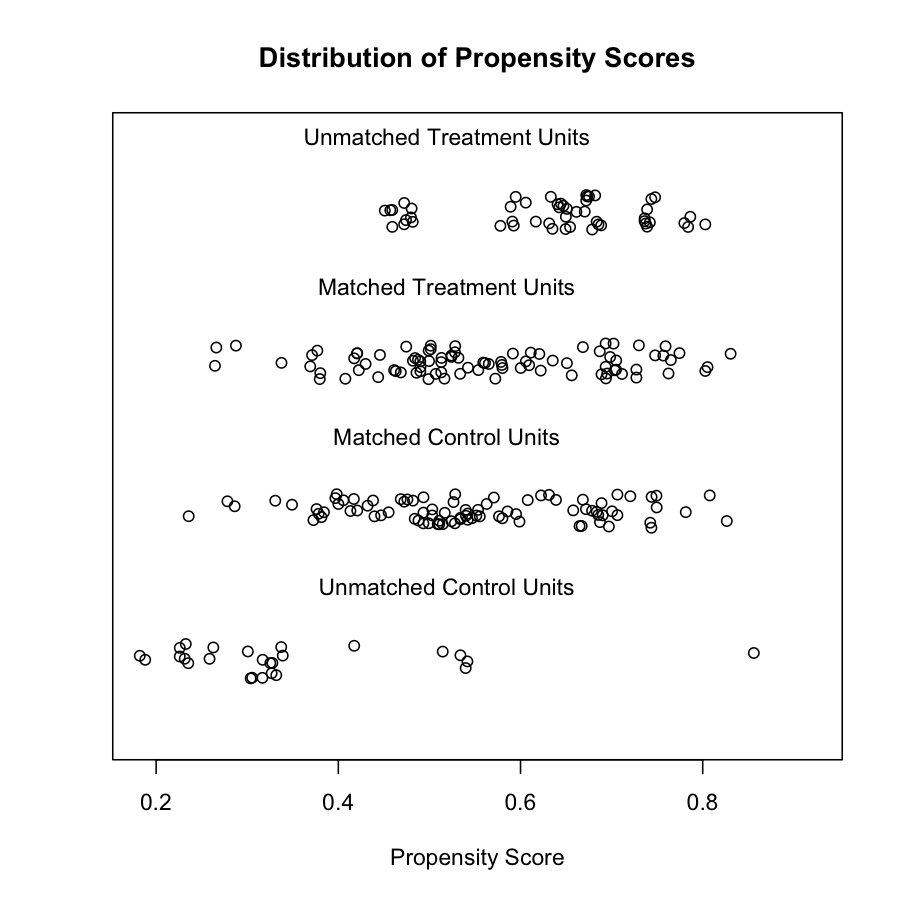


**Supplementary figure 3**: Line-plot of standardized differences by propensity score-matching analysis in patients with COVID-19


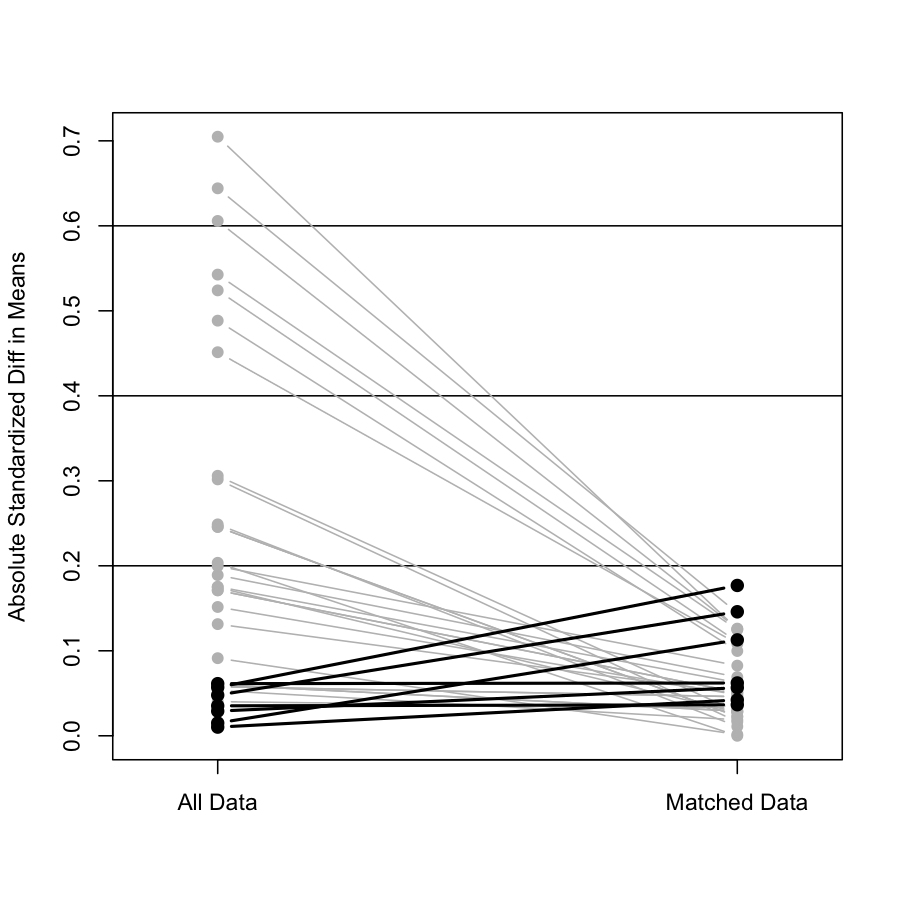


**Supplementary Figure 4**: The survival rate of patients with COVID-19 according to the presence of pulmonary thromboembolism (Kaplan-Meier survival analysis)


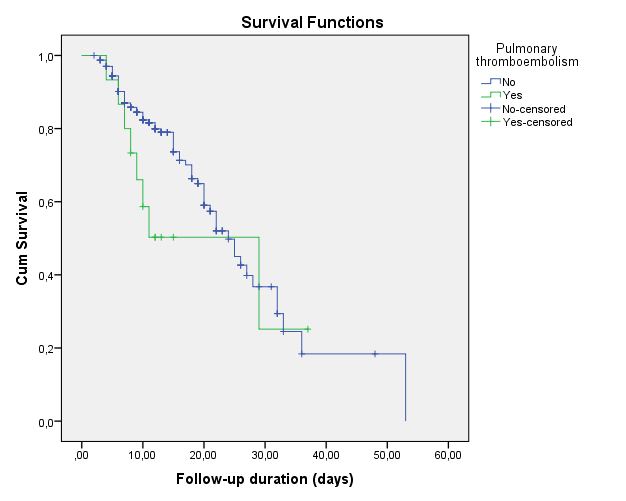


Log-Rank; p=0.15

**Supplementary Figure 5**: ROC curve of d-dimer levels for the development of any thromboembolic event in patients with COVID-19


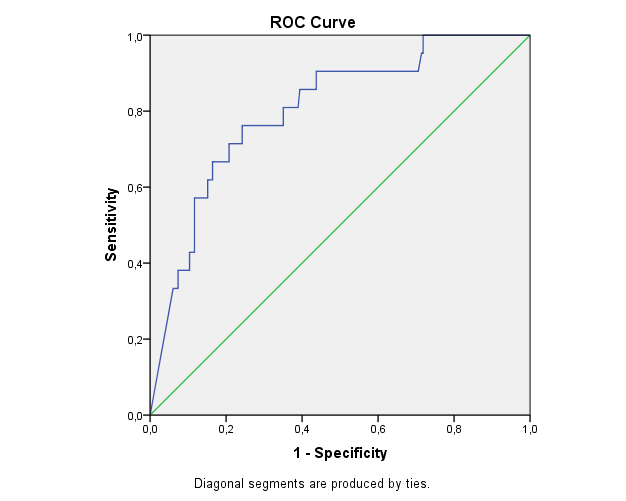


**Supplementary Figure 6**: ROC curve of d-dimer levels for the development of pulmonary thromboembolism in patients with COVID-19


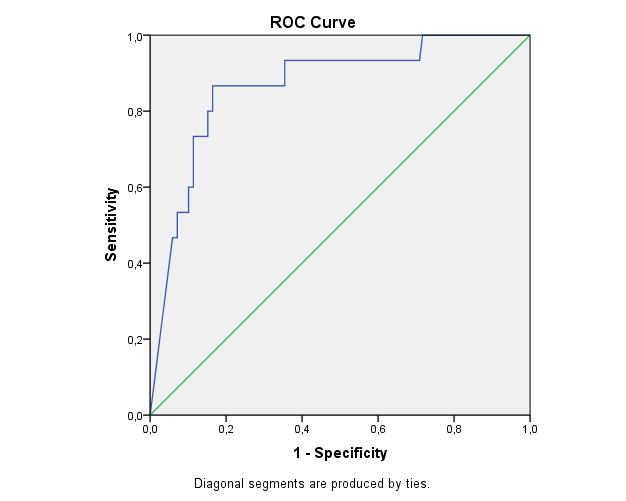


**Supplementary Figure 7**: ROC curve of d-dimer levels for the development of acute coronary syndrome in patients with COVID-19


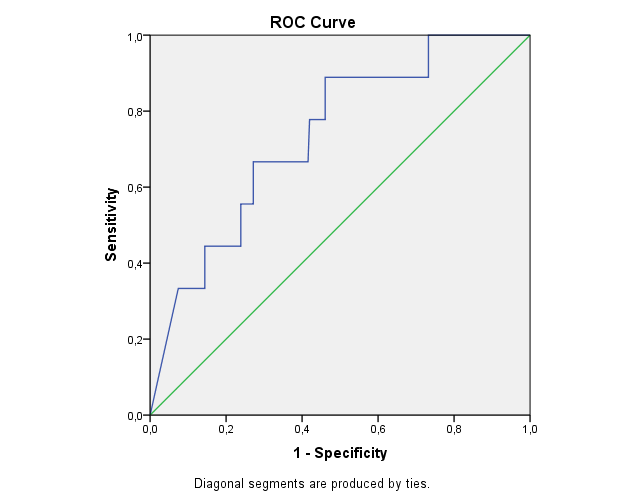


**Supplementary Figure 8**: ROC curve of mcHIS score for the development of any thromboembolic event in patients with COVID-19


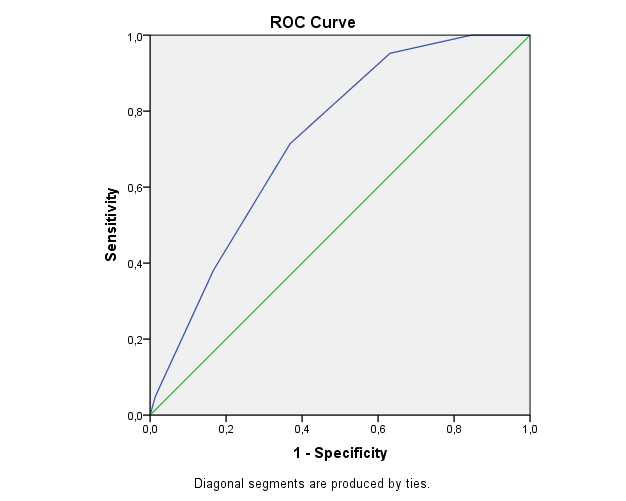


**Supplementary Figure 9**: ROC curve of mcHIS score for the development of pulmonary thromboembolism in patients with COVID-19


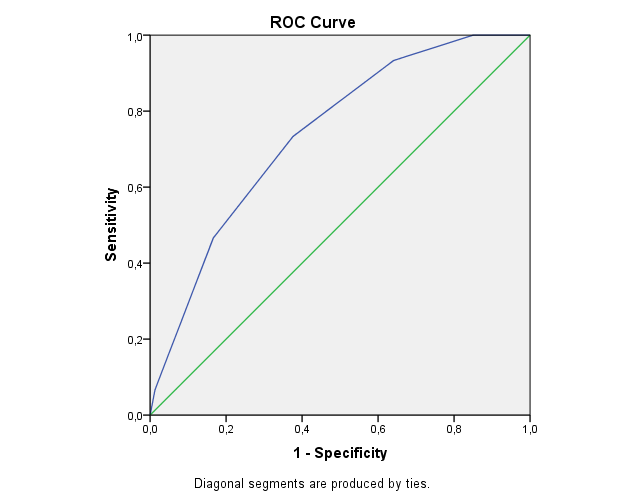


**Supplementary Figure 10**: ROC curve of mcHIS score for the development of acute coronary syndrome in patients with COVID-19


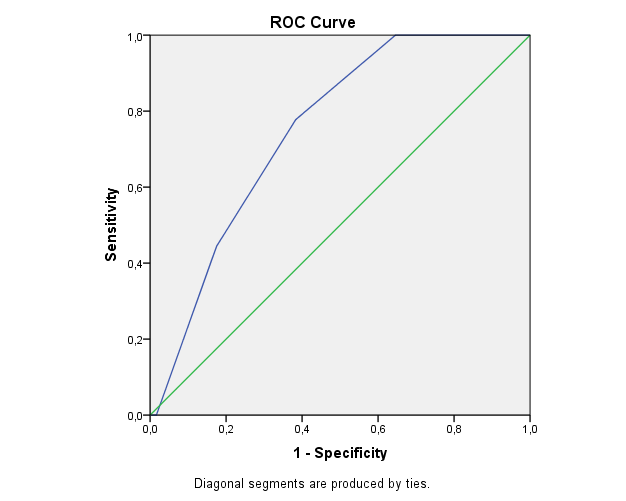


**Supplementary Figure 11**: ROC curve peak levels of CRP for the development of any thromboembolic event in patients with COVID-19


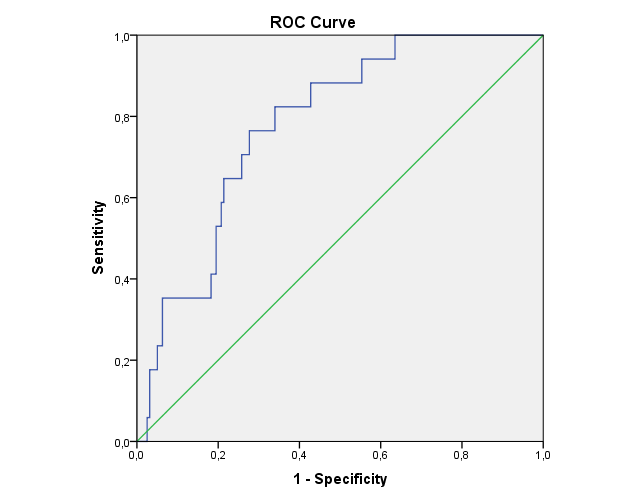


**Supplementary Figure 12**: ROC curve peak levels of CRP for the development of pulmonary thromboembolism in patients with COVID-19


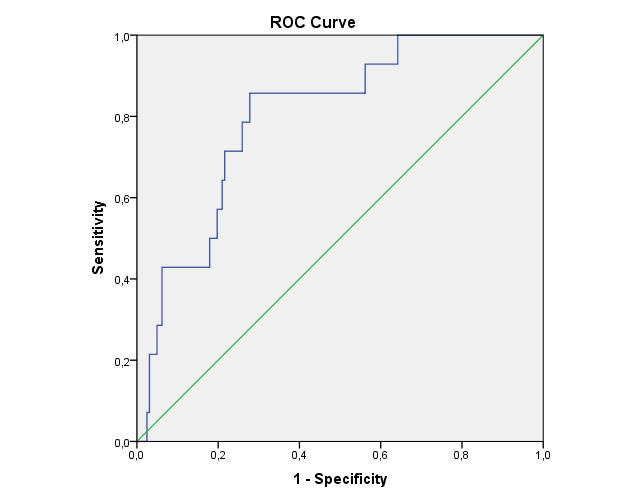


**Supplementary Figure 13**: ROC curve peak levels of CRP for the development of acute coronary syndrome in patients with COVID-19


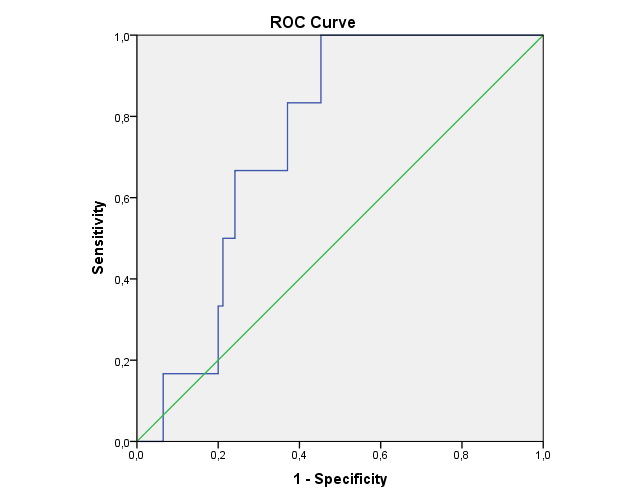


**Supplementary Figure 14**: ROC curve peak levels of LDH for the development of acute coronary syndrome in patients with COVID-19


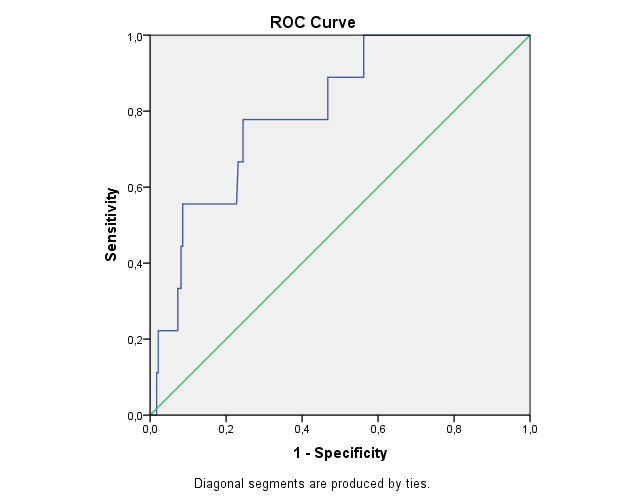


**Supplementary Figure 15**: ROC curve peak levels of LDH for the development of any thrombotic event in patients with COVID-19


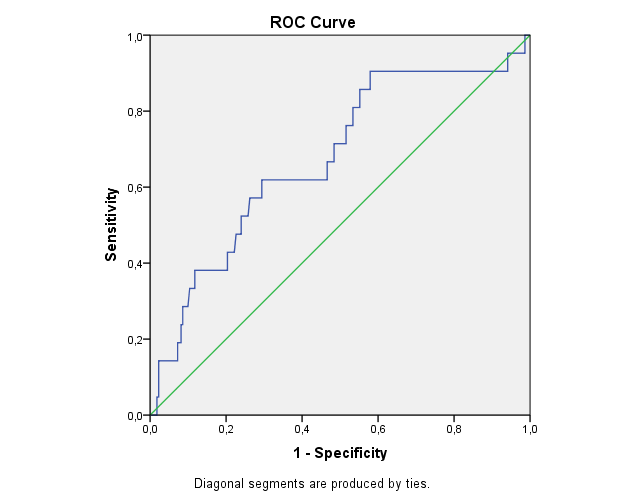

Supplement: Supplementary file 1 — Supplementary Information. [file 41598_2024_62079_MOESM1_ESM.docx]
